# Experimental data for the characterization of heat transfer processes in a cement based thermal energy storage system with helical heat exchanger

**DOI:** 10.1016/j.dib.2019.104721

**Published:** 2019-11-05

**Authors:** Johannes Nordbeck, Sebastian Bauer, Christof Beyer

**Affiliations:** Institute of Geosciences, University of Kiel, Kiel, Germany

**Keywords:** Heat storage, Helical heat exchanger, Lab scale experiment, Storage characterization, Temperature distribution, Model verification

## Abstract

This document compiles the detailed experimental data and description of four different heat charging tests presented in Nordbeck et al. ([1]), which aimed at the basic performance characterization of a lab-scale prototype of a new scalable, cement based, sensible heat storage system. The data set contains transient distributed measurements of temperatures within the storage as well as measurements of the experimental boundary conditions (heat carrier fluid flow rates, charging and laboratory temperatures) at high temporal resolution. In addition, the geometrical configuration of the storage and its component parts as well as the associated thermal material parameters are specified. The presented data is useful to assess and compare storage characteristics (storage capacities, charging/discharging rates, energy efficiency, heat loss behaviour) of the new heat storage system. The data can also be used as a reference data set for the development and verification of numerical models of modular solid-liquid heat storages or other related geothermal systems such as ground source heat pumps or energy piles using helical heat exchangers.

**Table:** Specifications Table

Subject	Renewable Energy, Sustainability and the Environment
Specific subject area	Sensible Thermal Energy Storage
Type of data	Table Graph Figure
How data were acquired	Temperature data was measured by K-type thermocouples (National Instruments) and logged using Labview; Flow rate data was measured by magnetic inductive flow meter (Ifm) and logged by HOBO datalogger and HOBOware software
Data format	Raw and processed
Parameters for data collection	Temperature data was recorded in Kelvin and converted to °C within Microsoft Excel.
Description of data collection	Temperature and flow meter data was logged in 1 minute increments using LabVIEW 2017 software; Logging of flux meter data was performed with HOBO datalogger and HOBOware software;
Data source location	Institution: Geotechnikum, Institute for Geoscience, University of Kiel, Kiel, Germany
Data accessibility	Descriptions, graphs, figures, geometrical data and parameter tables are provided with the article, measurement data time series are hosted in a public repository. Repository name: Mendeley Data https://doi.org/10.17632/5yv9gznh3x.2 Direct URL to data: https://doi.org/10.17632/5yv9gznh3x.2#file-7101a55f-8046-4aab-b8a4-1c98955fba18
Related research article	Johannes Nordbeck, Sebastian Bauer, Christof Beyer Experimental characterization of a lab-scale cement based thermal energy storage system Applied Energy 256 (2019) 113937 https://doi.org/10.1016/j.apenergy.2019.113937

**Table:** 

**Value of the Data** • The presented experimental data is useful to assess and compare the storage characteristics (storage capacities, charging/discharging rates, energy efficiency and heat loss behaviour) of a new modular sensible heat storage system. • The data is interesting for the construction/geotechnical engineers as well as a scientific audience working on thermal energy storage technologies. • The data can be used as a basis for the development and verification of detailed numerical models of the new modular heat storage system, which are necessary for efficient system optimization and sensitivity analyses. • The experimental data are potentially useful also as a reference data set for the development and verification of numerical models for other related heat storage or geothermal systems such as ground source heat pumps or energy piles using helical heat exchangers.

## 1 Data

**Fig. 1:**
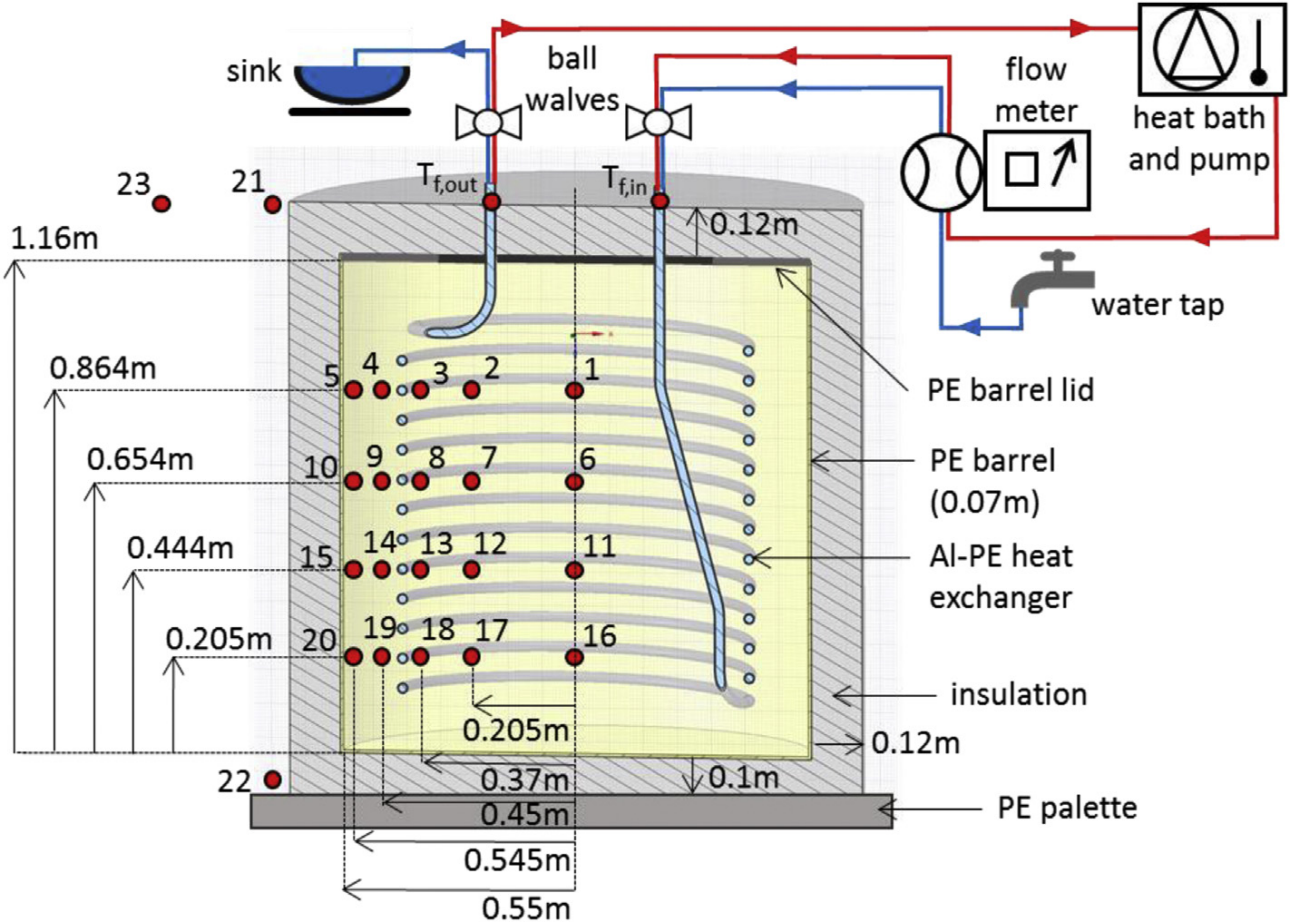
Sketch of the experimental setup with positioning of temperature sensors T_1_ - T_20_ inside the storage matrix, sensors T_21_ - T_23_ outside of the storage unit, two sensors inside the heat exchanger inlet and outlet pipes (T_f,in_, T_f,out_), heat charging (red) and discharging (blue) circuits, and storage component parts.

Fig. 1 shows the lab-scale prototype unit of the proposed modular solid-liquid heat storage system and the experimental setup that was used to investigate its storage characteristics. The system consists of a 1 m³ plastic barrel with a helical heat exchanger installed inside. The storage medium is a fully water saturated cementitious material with a high porosity. The storage unit is covered by insulation from all sides to minimize impacts from a fluctuating ambient temperature and placed on a PE palette. The charging cycle uses a heat bath with a pump as heat source, while the discharging cycle uses cold tap water as working fluid. Dimensions of the storage units component parts and positions of temperature sensors are included.

**Fig. 2:**
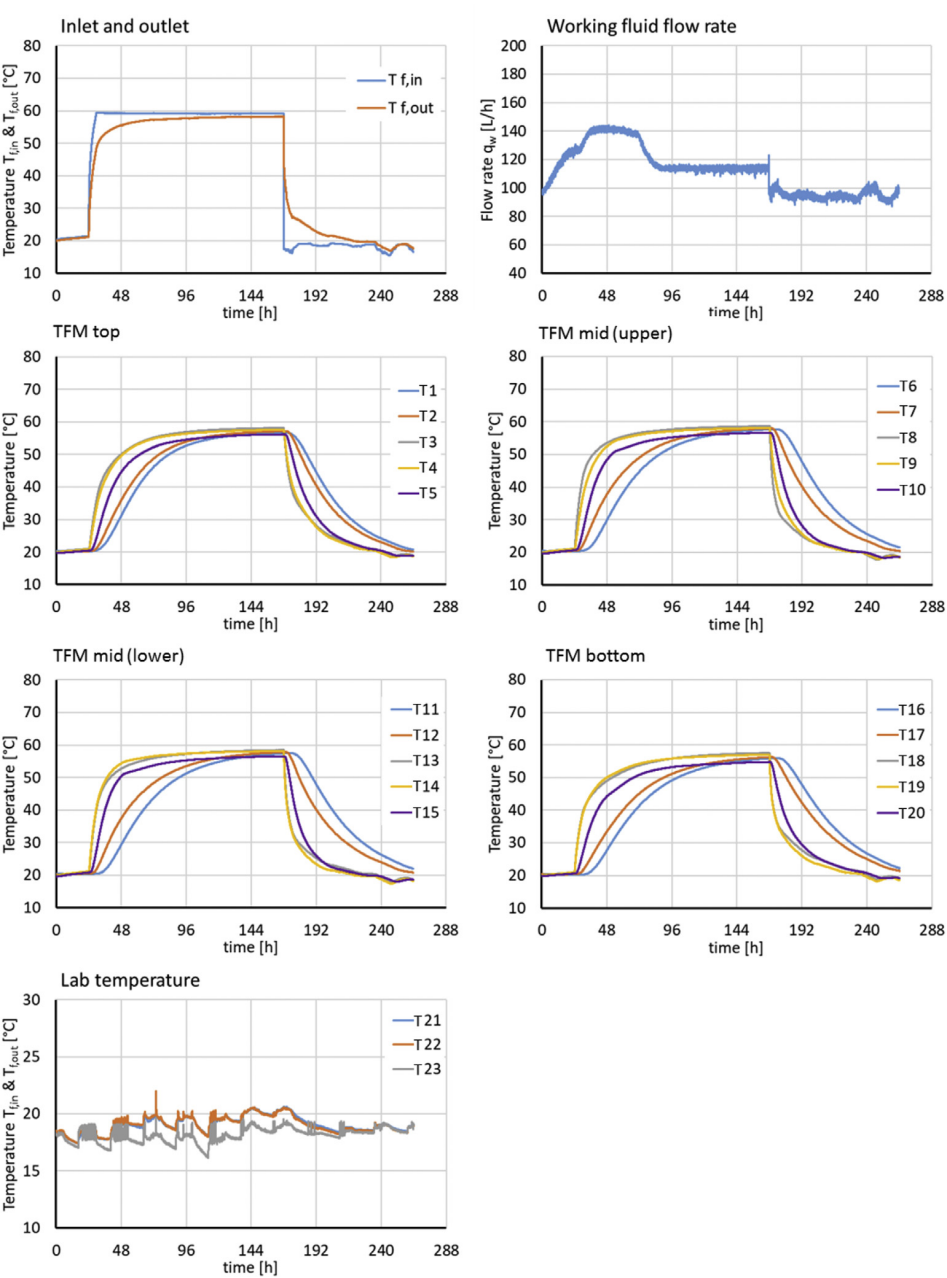
Experimental boundary conditions (T_f,in_, fluid flow rate, temperatures at laboratory temperature sensors T_21_ - T_23_) and monitoring data (T_f,out_, temperatures at sensors T_1_ - T_20_ inside the TFM) for experiment E1.

**Fig. 3:**
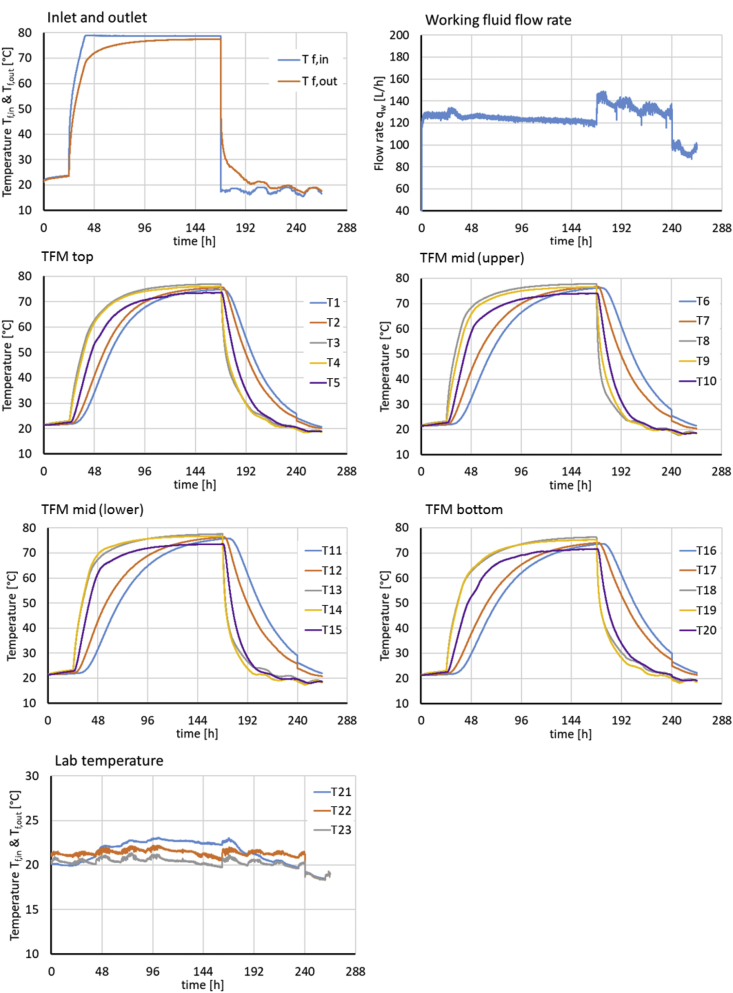
Experimental boundary conditions (T_f,in_, fluid flow rate, temperatures at laboratory temperature sensors T_21_ - T_23_) and monitoring data (T_f,out_, temperatures at sensors T_1_ - T_20_ inside the TFM) for experiment E2.

Fig. 2, Fig. 3 present the experimental boundary conditions (*T*_*f,in*_, fluid flow rate, temperatures at laboratory temperature sensors T21-T23) and monitoring data (*T*_*f,out*_, temperatures at sensors T1-T20 inside the TFM) for the charging/discharging experiments E1 and E2. Both experiments start with 24h of de-aeration, by circulating water at ambient temperature through the system. This is followed by 144h of charging at 60 and 80 °C *T*_*f,in*_ for E1 and E2, respectively. During charging temperatures at sensors T1-T20 inside the TFM increase. During the following discharge temperatures at T1-T20 decrease again, starting at locations near the heat exchanger, while the storage centre retains a higher temperature.

**Fig. 4:**
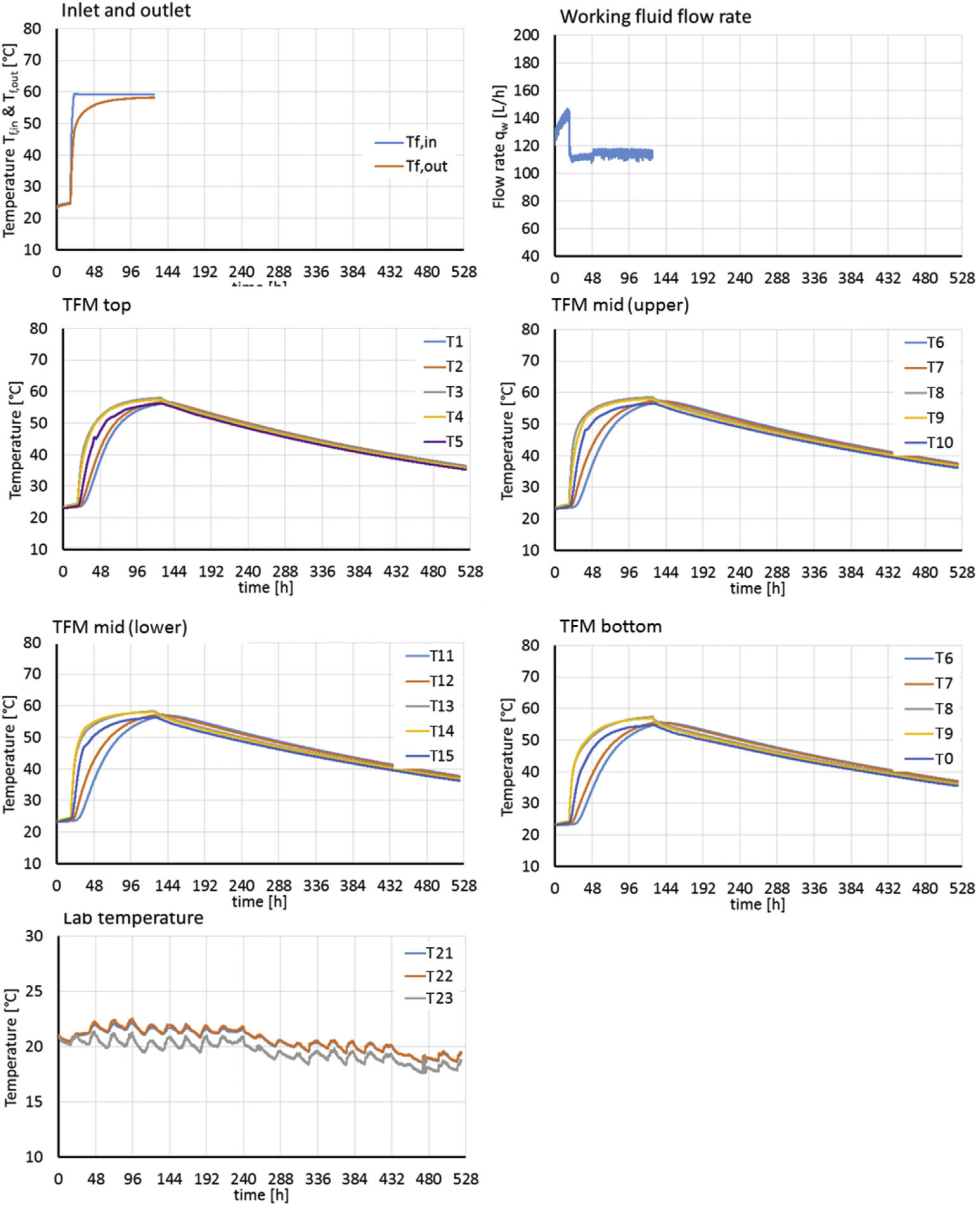
Experimental boundary conditions (T_f,in_, fluid flow rate, temperatures at laboratory temperature sensors T_21_ - T_23_) and monitoring data (T_f,out_, temperatures at sensors T_1_ - T_20_ inside the TFM) for experiment E3. The working fluid was circulated through the heat exchanger only between 0 and 168 h.

**Fig. 5:**
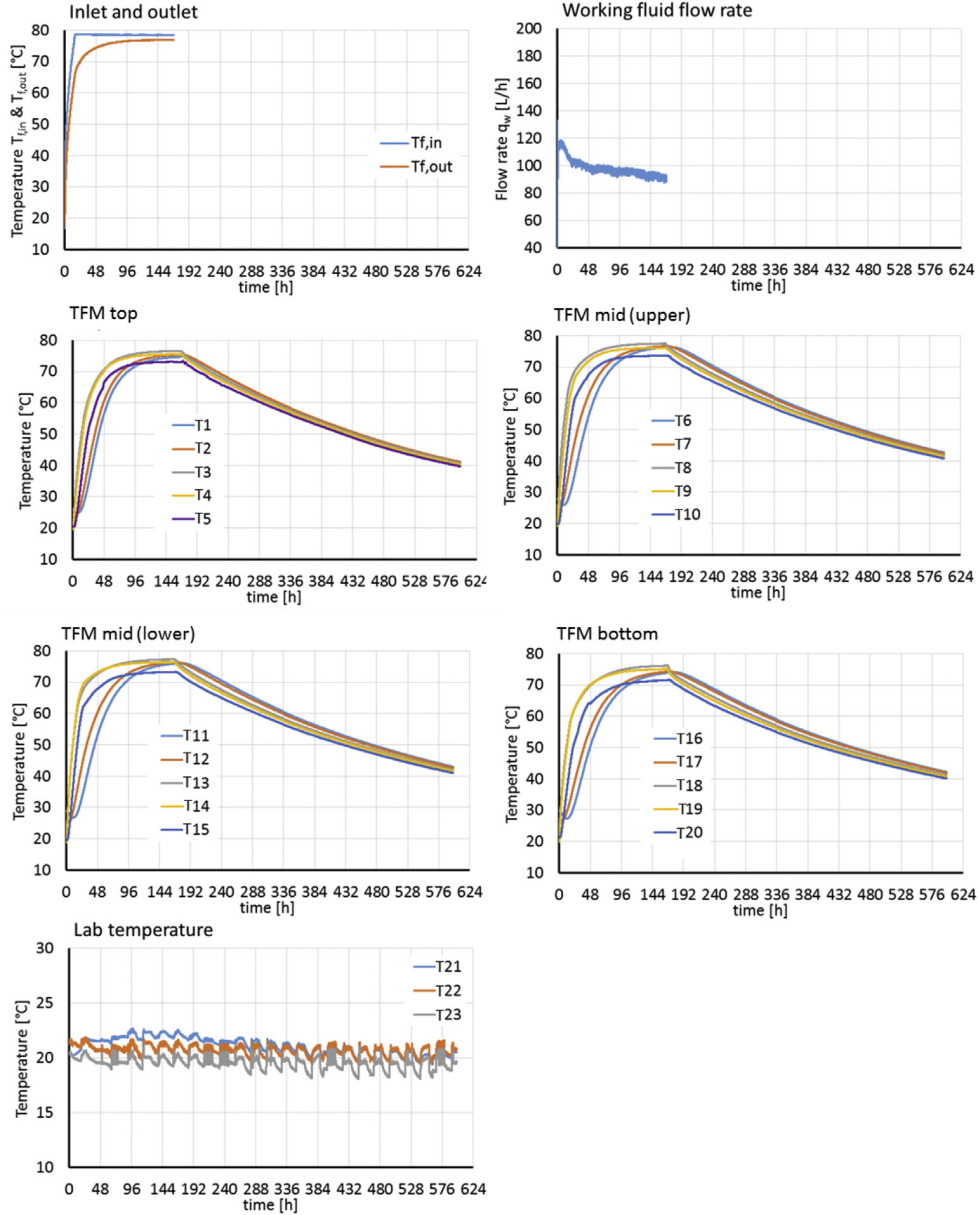
Experimental boundary conditions (T_f,in_, fluid flow rate, temperatures at laboratory temperature sensors T_21_ - T_23_) and monitoring data (T_f,out_, temperatures at sensors T_1_ - T_20_ inside the TFM) for experiment E4. The working fluid was circulated through the heat exchanger only between 0 and 168 h.

Fig. 4, Fig. 5 present the experimental boundary conditions (*T*_*f,in*_, fluid flow rate, temperatures at laboratory temperature sensors T21-T23) and monitoring data (*T*_*f,out*_, temperatures at sensors T1-T20 inside the TFM) for the charging/passive cooling experiments E3 and E4. After de-aeration of the system, both experiments start with charging at 60 (E3) and 80 °C (E4) until constant temperatures are reached inside the TFM and for *T*_*f,out*_. Passive cooling of the storage unit follows the initial charging phase where temperatures slowly decrease inside the TFM, starting with the edges, over a period of 396 (E3) and 430 h (E4).

Table 1 presents the geometric specifications and thermal properties of all components parts materials of the experimentally investigated laboratory prototype heat storage unit, as determined by own measurements or from manufacturer data sheet specifications. Table 2 presents the exact positions of K-type thermocouple temperature sensors within and outside of the storage unit and Table 3 presents the experimental schedules of storage experiment E1, E2, E3 and E4.

**Table 1:** Materials, thermal properties, dimensions and geometrical specifications of the experimental heat storage unit.

**Barrel**		
material	polypropylene	
density	950 kg/m³	
thermal conductivity	0.4 W/m/K	
volumetric heat	1.824 MJ/m³/K	
inner/outer diameter	110/111.4 cm	
barrel-insulation interface area	6.07 m^2^	
Height	116 cm	from inside bottom to lid
mantle wall thickness	0.7 cm	
bottom thickness	1 cm	
**barrel lid**		consists of two parts: inner and outer lid
material	polypropylene	
density	950 kg/m³	Density
thermal conductivity	0.4 W/m/K	thermal conductivity
volumetric heat	1.824 MJ/m³/K	volumetric heat
barrel lid-insulation interface area	0.97 m^2^	
inner lid diameter	56 cm	Disk shaped
inner lid thickness	3 cm	
outer lid inner/outer diameter	50/111.4 cm	annulus shaped
outer lid thickness	0.7 cm	
**tubular HHX**		
material	Al-PE composite	helical shape, Wavin GmbH
Density	1515 kg/m³	Density
thermal conductivity	0.4 W/m/K	thermal conductivity
volumetric heat	1.590 MJ/m³/K	volumetric heat
HHX-TFM interface area	2.59 m^2^	
pipe inner/outer diameter	2.0/2.5 cm	
HHX diameter (pipe center to pipe center)	82.0 cm	
pitch height	7.0 cm	
HHX height	91 cm	from bottom of lowest to top of uppermost coil
HHX height above bottom	7 cm	from TFM bottom to bottom of lowest coil
total pipe length within TFM	33.5 m	
total pipe volume within TFM	16.44 L	
pipe length to T_f,in_, T_f,out_	50 cm	from barrel lid to sensor in inlet/outlet pipe
**TFM**		
material	Füllbinder L, water	Schwenk Zement KG
density	2276 kg/m³	Density
thermal conductivity	0.96 W/m/K	Measured at 20 °C laboratory temperature
volumetric heat	3.423 MJ/m³/K	volumetric heat
porosity	0.543	
TFM-barrel interface area	5.91 m^2^	
volume	0.97 m³	
filling height	105.0 cm	
**bottom insulation**		
material	Styrodur	
density	50 kg/m³	
thermal conductivity	0.034 W/m/K	
bottom insulation – lab interface area	1.44 m^2^	
thickness	10.0 cm	
**mantle & lid insulation**		
material, layer 1	Armaflex	Armacell International S.A.
thickness	2 cm	
density	50 kg/m³	
thermal conductivity	0.033 W/m/K	
material, layer 2	Eco-Skin	Austria Email AG
thickness	10 cm	
mantle & lid insulation – lab interface area	7.38m^2^	
density	50 kg/m³	
thermal conductivity	0.038 W/m/K	
**tube insulation**		
Material	Armaflex	Armacell International S.A.
density	50 kg/m³	
thermal conductivity	0.033 W/m/K	
thickness	2 cm

**Table 2:** Temperature sensors and their positions within or around the experimental heat storage prototype. R indicates the radial distance from the center symmetry axis of the storage, h the height above the surface of barrel bottom wall.

sensor ID	measurement location	r [cm]^a^	h [cm]^b^
T1	TFM top center	0	86.4
T2	TFM top outer center	20.5	86.4
T3	TFM top HX inside	37	86.4
T4	TFM top HX outside	45	86.4
T5	TFM top wall	54.5	86.4
T6	TFM upper half center	0	65.4
T7	TFM upper half outer center	20.5	65.4
T8	TFM upper half HX inside	37	65.4
T9	TFM upper half HX outside	45	65.4
T10	TFM upper half wall	54.5	65.4
T11	TFM lower half center	0	44.4
T12	TFM lower half outer center	20.5	44.4
T13	TFM lower half HX inside	37	44.4
T14	TFM lower half HX outside	45	44.4
T15	TFM lower half wall	54.5	44.4
T16	TFM bottom center	0	16.4
T17	TFM bottom outer center	20.5	16.4
T18	TFM bottom HX inside	37	16.4
T19	TFM bottom HX outside	45	16.4
T20	TFM bottom wall	54.5	16.4
Tf,in	0.5 m from inlet		
Tf,out	0.5 m from outlet		
T21	laboratory, 0 m from storage unit (at storage top)		
T22	laboratory, 0 m from storage unit (at storage bottom)		
T23	laboratory, 2 m from storage unit (at 1 m height from lab floor)		

a Radial distance from center axis.

b Height above TFM bottom.

**Table 3:** Schedules and boundary conditions of experiments E1, E2, E3 and E4.

Experiment	equilibration & deareation [h]	charging duration [h]	charging temperature^a^ [°C]	discharging duration [h]	discharging temperature^b^ [°C]	passive cooling duration [h]
E1	24	144	60	96	15.43–19.21	–
E2	24	144	80	72	16.18–19.17	–
E3	24	126	60	–	–	396
E4	24	168	80	–	–	430

a Heat bath temperature.

b Variable tap water temperature.

## 2 Experimental design, materials, and methods

The data compiled in this document was collected during the experimental investigation of a laboratory scale prototype module of a new scalable, cement based sensible heat storage system presented in Ref. [1]. Four different experimental data sets are presented here, complemented by a detailed description of the specifications of the prototype heat storage system and the laboratory experimental setup, experimental schedules and boundary conditions. The storage system is suited for storage temperatures of up to 90 °C, and heat exchange is achieved using an embedded helix type heat exchanger. The storage matrix consists of solid porous cement and is fully saturated with pore water in order to increase the energy density and thus the storage capacity of the system. All components of the storage system consist of commercially available standard materials. Arrays of storage modules can be installed into the subsurface next to or below a building, in cellars or in designated storage spaces, and be tailored to match spatial constrictions of the specific application case. The cementous storage material has a high mechanical stability and the systems thus can also serve as building foundation. The prototype module investigated by Nordbeck et al. [1] was constructed at the Geotechnical Center (“Geotechnikum”) of the Institute for Geoscience, Christian-Albrechts University Kiel (Germany) between June and September in 2016, and the storage performance was characterized in terms of storage capacity, achievable heat charging and discharging rates as well as the heat loss using the set of four storage experiments performed between September and November 2016 (E1 & E3) and from November to December 2017 (E2 & E4) and described here in full detail.

The construction of the heat storage prototype unit in the Geotechnikum allowed the performance of the storage experiments in a well controlled environment, i.e. under only slightly varying lab temperature and extensive monitoring. The storage unit was constructed by installing a tubular helical heat exchanger in a porous, water saturated thermal filling material contained by a polypropylene (PP) barrel, which is covered by a lid (Fig. 1). The barrel has the following geometrical specifications: inner diameter = 110 cm, height = 116 cm, volume = 1.1 m³, bottom thickness = 1 cm, mantle wall thickness = 0.7 cm. It is placed on a 10 cm sheet of Styrodur (BASF [2]) as the bottom insulation, which in turn is placed on a heavy duty polyethylene (PE) pallet. The lid and mantle surfaces of the storage unit were insulated by 2 cm of Armaflex elastomer (Armacell International S.A. [3]) and 10cm of Eco-Skin (Austria Email AG [4]), an polyester fibre mat covered by a 1mm coating of PE on the outside. The lid consists of an inner PP disk (56 cm diameter) and an outer PP hollow ring (111.4 cm outer diameter, 50 cm cut out diameter). The disk can be tightened onto the ring with 18 steel bolts. The inlet and outlet pipes of the heat exchanger protrude from two ducts inside the inner disk and three additional ducts (2.5 cm diameter) were drilled into the lid for sensor cables.

The helical heat exchanger (7 cm height of lowest loop above the bottom of the barrel, 91 cm total height (lowermost to topmost loop), 82 cm diameter, 7 cm pitch (distance between tube centers of two loops), 33.5 m total pipe length within the storage unit) consists of an aluminum-PE composite pipe (Wavin PE-Xc [5], 2 cm inner diameter, 2.5 cm outer diameter, 2.5 mm wall thickness). Water without additives is circulated as a heat carrier fluid through the heat exchanger. It is coiled and held in place and shape by a slim frame on the inside of the helix. Wooden spacers are clamped onto the bottom and top of the frame, which keep the heat exchanger centered within the storage unit. The thermal filling material (TFM) consists of Füllbinder L (Schwenk Zement KG [6]), which is a commercially available cement-based filling and sealing material with high weight fractions of calcite (>60%), quartz (ca. 10%) and hydraulic binder (30%) [7]. The water cement mixture was prepared in a weight ratio of 0.8 using a high-speed colloidal mixer and filled into the barrel with the kind help of Bau-ABC Rostrup. After a curing time of 30 days, the TFM has a high water filled porosity of 0.543 and fills the barrel up to a height of 105cm. Considering the volume of the heat exchanger and its frame (approximately 30 L), the TFM volume is 0.97 m³ with an estimated error of ± 1%, which equals ± 1cm of height of the TFM inside the barrel. 30 L of water are added on top of the TFM, to keep it from drying out and ensure a full saturation of the pore space. The remaining 0.1 m³ above the TFM is filled by air and water vapour. The geometrical specifications of all component parts are summarized in Table 1.

The thermal properties of the TFM were measured by Miao et al. [8] on three small samples (5cm diameter, 10cm length) with a Decagon KD2 Pro thermal needle probe transient line-source measurement technique in accordance to ASTM D5334-08 [9] and IEEE 442 standards [10]. The needle probes TR-1 (single needle) and SH-1 (dual needle) measured the thermal conductivity and volumetric heat of fully saturated samples at an average laboratory temperature of 20 °C and atmospheric pressure. The KD2 Pro analyzer is accurate within ± 10% inside a range of 0.2–4W/m/K for conductivity and ± 10% for volumetric specific heat at conductivities above 0.1 W/m/K [11]. Density and porosity of the TFM were determined by gravimetric measurements. Thermal properties and densities of the HHX Al-PE composite pipe, PP-barrel, and the different insulation layers were considered as indicated on manufacturer data sheets ([[2], [3], [4], [5]]). These properties are summarized and included in Table 1. In addition to the bulk thermal properties of the material groups, the heat transfer behavior of the whole storage unit is controlled by the heat transfer across the HHX-TFM interface during charging and discharging and by the heat transfer across the insulation-lab interface as heat loss from the storage unit into the lab environment. Nordbeck et al. [1] present an extensive analysis of the heat transfer behavior across these interfaces and the thermal storage characteristics of the whole system, with a heat balance model and the experimental data presented in this article.

A total of 20 K-type thermocouple temperature sensors numbered T_1_ to T_20_ (National Instruments) were placed within the storage unit in four radial transects before the filling with the TFM in order to record temperatures during the storage experiments at the center, between center and heat exchanger, directly next to the heat exchanger (inwards and outwards) and near the inner wall of the barrel, covering the vertical extent of the storage unit. The sensors were fixed on cords, which were spanned between the wooden spacers. Two additional thermocouples (T_f,in_, T_f,out_) were installed inside the inlet and outlet pipes to measure the supply and return flow temperatures. Three thermocouples (T_21_-T_23_) measure the laboratory room temperature at increasing distances to the storage unit (xyz m). The measurement locations are shown in Fig. 2 and are detailed in Table 2. All temperature sensors are connected to a data acquisition device (National Instruments). The estimated temperature measurement error is ±0.45 °C for the thermocouples [12].

Heat source and pump for the heat carrier fluid are combined in a heat bath system (CC–215B, Huber Kältemaschinenbau GmbH [13]), which consists of a heater, a 15 L hot water reservoir and a suction pump with a combined power of 2kW, which provides constant supply temperatures and flow rates between 100 and 200L/h. Rubber hoses with inner and outer diameters of 9 and 16mm connect the inlet and outlet pipes of the heat exchanger with the heat bath system to complete the thermal charging cycle. The distance between hot water source and heat storage inlet is 4 m. To minimize heat losses, all hoses and pipes that are part of the charging cycle were insulated with Armaflex elastomer tubes with a wall thickness of 2cm. For extracting heat energy previously stored in the storage unit, two ball valves are turned to direct cold water from a nearby water tap into the inlet and the return flow from the outlet into a nearby sink. A magnetic inductive flowmeter (model SM6004, ifm electronic GmbH [14]) measures and records the water flow rate with an accuracy of ± 2% (ifm electronic, 2015) and at 1 minute intervals.

The data sets of four experiments are presented here in total (cf. Table 3). In experiments E1 and E2, two thermal charging and discharging tests were performed on the storage unit in order to determine the basic storage characteristics of the heat storage system, i.e. storage capacities and heat charging/discharging rates over time. The first experiment (E1) was performed at 60 °C supply temperature of the heating bath during the charging phase, the second experiment (E2) was performed at 80 °C supply temperature. The experiments started with a 24h period of de-aeration by circulating water through the system at laboratory temperature *T*_*l*_ in order to purge the air from the heat exchanger. After 24 h the heater was activated. Constant inlet temperatures *T*_*f,in*_ of 59.3 and 78.9 °C were reached after 6 and 16h of heating-up, respectively, and kept constant for 6 days of heat charging the storage unit in order to reach stationary storage temperatures *T*_*s*_. The charging phase in each experiment was immediately followed by the heat discharging phase with cold tap water over a period of 4 days (E1) and 3 days (E2), respectively. During the discharging phase, *T*_*f,in*_ fluctuates between 15.43 and 19.21 °C due to the variability in the water supply temperature of the university water system.

During the experiments, flow meter and temperature data at all sensors were logged in 1 minute intervals in units of m³/s (flow rate and K temperature). The data was imported from the log files to excel and converted to units of L/h and °C, respectively. All figures provided with this document are based on this data.

Fig. 2, Fig. 3 give an overview of the experimental boundary conditions (i.e. the fluid inlet and laboratory temperatures *T*_*f,in*_ and *T*_*21*_ *- T*_*23*_), and the flow rate of the working fluid (*q*_*w*_) and monitoring results (fluid return temperature *T*_*f,out*_ and TFM temperatures at all sensor locations *T*_*1*_ *- T*_*20*_) for experiments E1 abd E2, respectively.

Experiments E3 and E4 were performed in order to analyze the heat loss behaviour of the storage unit over time. As E1 and E2, both experiments started with a 24h period of de-aeration by circulating water through the system at laboratory ambient temperature. After 24 h the heater was activated. Constant inlet temperatures *T*_*f,in*_ of 59.16 (E3) and 78.47 °C (E4) were reached after 126 and 168 h of heating-up, respectively, and kept constant for 6 days of heat charging the storage unit in order to reach stationary storage temperatures *T*_*s*_. Heat bath and pump system then were deactivated and the storage unit was left to cool down passively, i.e. without further circulation of a working fluid.

Fig. 4, Fig. 5 give an overview of the experimental boundary conditions (i.e. the fluid inlet and laboratory temperatures *T*_*f,in*_ and *T*_*21*_ *- T*_*23*_, and the flow rate of the working fluid *q*_*w*_) and monitoring results (fluid return temperature *T*_*f,out*_ and TFM temperatures at all sensor locations *T*_*1*_ *- T*_*20*_) for experiments E3 and E4, respectively.
